# Genetic diversity and population structure of indigenous chicken of Bangladesh using microsatellite markers

**DOI:** 10.5713/ajas.20.0189

**Published:** 2020-08-03

**Authors:** Muhammad Abdur Rashid, Prabuddha Manjula, Shakila Faruque, A. K. Fazlul Haque Bhuiyan, Dongwon Seo, Jahangir Alam, Jun Heon Lee, Mohammad Shamsul Alam Bhuiyan

**Affiliations:** 1 Department of Animal Breeding and Genetics, Bangladesh Agricultural University, Mymensingh-2202, Bangladesh; 2 Poultry Production Research Division, Bangladesh Livestock Research Institute, Dhaka-1341, Bangladesh; 3 Division of Animal and Dairy Science, Chungnam National University, Daejeon 34134, Korea; 4 Animal Biotechnology Division, National Institute of Biotechnology, Dhaka-1349, Bangladesh

**Keywords:** Indigenous Chicken, Genetic Diversity, Microsatellite Marker, Bangladesh

## Abstract

**Objective:**

The objectives of this study were to investigate the genetic diversity, population structure and relatedness among the five chicken populations of Bangladesh using microsatellite markers.

**Methods:**

A total of 161 individuals representing 5 chicken populations (non-descript Deshi [ND], naked neck [NN], hilly [HI], Aseel [AS], and red jungle fowl [JF]) were included in this study to investigate genetic diversity measures, population structure, genetic distance and phylogenetic relationships. Genotyping was performed using 16 selected polymorphic microsatellite markers distributed across 10 chromosomes.

**Results:**

The average observed and expected heterozygosity, mean number of alleles and polymorphic information content were found to be 0.67±0.01, 0.70±0.01, 10.7 and 0.748, respectively in the studied populations. The estimated overall fixation index across the loci (F), heterozygote deficiency within (F_IS_) and among (F_IT_) chicken populations were 0.04±0.02, 0.05 and 0.16, respectively. Analysis of molecular variance analysis revealed 88.07% of the total genetic diversity was accounted for within population variation and the rest 11.93% was incurred with population differentiation (F_ST_). The highest pairwise genetic distance (0.154) was found between ND and AS while the lowest distance was between JF and AS (0.084). Structure analysis depicted that the studied samples can be categorized into four distinct types or varieties (ΔK = 3.74) such as ND, NN, and HI where AS and JF clustered together as an admixed population. The Neighbor-Joining phylogenetic tree and discriminant analysis of principal component also showed close relatedness among three chicken varieties namely AS, HI, and JF.

**Conclusion:**

The results reflected that indigenous chicken of Bangladesh still possess rich genetic diversity but weak differentiation among the studied populations. This finding provides some important insight on genetic diversity measures that could support the designing and implementing of future breeding plans for indigenous chickens of Bangladesh.

## INTRODUCTION

Poultry takes a vital place in global food chain. In fact, poultry is an integral part of rural households in the least developed countries around the world. There are about 255.31 million chickens in commercial and subsistence production systems in Bangladesh [1]. Among poultry, the chicken population is almost 90%, followed by ducks (8%) and rest 2% occupied by quail, pigeon, geese, and others [2]. The national share of commercial strains of chicken and indigenous family poultry in terms of egg production is almost equal (50:50) and that of meat production is 60:40 [3].About 89% of rural households keep indigenous chicken with an average flock size of 5.33 per holding under a backyard scavenging system that reflects the significance of indigenous chicken in Bangladesh perspective [4,5]. All elites, most city dwellers and some village level customers appreciate egg and meat produced from indigenous (*deshi*) chicken reared in scavenging system because their products are produced organically.

Indigenous chickens are lower in productivity but are well adapted to adverse tropical climate and fluctuating nutritional conditions compared to exotic chicken. However, the preferences for free-range indigenous chicken might be attributed to typical pigmentation, taste, leanness, firmness, high protein content and suitability for special dishes [3,6]. The identification, collection, evaluation, and conservation of different genotypes are an insurance against future need for breeding [7]. In this regard, unselected random mated indigenous chickens are considered as a huge treasure of many known and unknown genotypes, which could be beneficial to provide valuable additional attributes to the future poultry production. According to Bhuiyan et al [5], non-descript Deshi (ND), naked neck (NN), hilly (HI), and Aseel (AS) chickens are noteworthy among the indigenous chicken genetic resources of Bangladesh. It is notable to mention that Bangladesh Livestock Research Institute (BLRI) has undertaken a planned and systematic native chicken breeding program since 2000 and has improved the productivity of three different native chicken varieties (ND, HI, and NN) [8]. The productivity of the said genotypes has increased remarkably over the generations through selective breeding on-station in comparison to existing indigenous chicken varieties. Based on the mitochondrial DNA sequence polymorphisms, Bhuiyan et al [5] suggested that Bangladeshi indigenous chickens still possessed abundant genetic diversity and have originated from multiple maternal lineages. Apart from this, genetic characterization of these varieties has not yet been sufficiently explored which is a prerequisite to knowing within and between population differentiation in order to establish them as breeds or varieties.

Microsatellites are widely used DNA markers for exploring genetic variation and phylogeny among populations of same species [9,10]. The usefulness of microsatellite (MS) markers in estimating genetic relatedness and diversity in chickens have been demonstrated in several indigenous breeds, inbred strains and in commercial chicken lines [11–14]. Although molecular genetic characterization of indigenous chicken of Bangladesh has been performed based on mitochondrial DNA, characterization using nuclear genomic DNA is absent until to date. Hence, it would be worthwhile to know the genetic diversity and population structures of available chicken varieties of Bangladesh for development of a proper conservation and breeding strategy. Here, we investigate three indigenous chicken varieties that have been maintained at BLRI along with red jungle fowl (JF) and AS chickens for assessing their genetic diversity, genetic distance and population structure using 16 highly polymorphic MS markers.

## MATERIALS AND METHODS

### Sample collection and DNA extraction

Institutional Animal Care and Use Committee (IACUC) reviewed the total experimental procedure and prior approval was obtained from IACUC for this experiment (Approval number: BLRI-PCUC-003). In total, 161 unrelated blood samples were collected from 5 different chicken populations of Bangladesh: ND (n = 41), NN (n = 44), HI (n = 39), AS (n = 18), and JF (n = 19). The Native Chicken Conservation and Improvement Project of BLRI maintains ND, NN, and HI flocks and these birds were used for sampling. Besides, blood samples of AS chicken were collected from Bangladesh Agricultural University (BAU) research flock as well as from the farmers of Brahmanbaria district. JF sampling was performed from their breeding habitats, Chittagong Hill Tract regions (22.20 °N and 92.35 °E). According to Bhuiyan et al [5], two different sub-species of JF are available in Bangladesh; *Gallus gallus gallus* and *Gallus gallus spadiceus*. However, blood samples were not categorized as per sub-species level for this study. All possible precautions were taken during sampling from unrelated individuals to prevent cross contamination. The blood samples were collected from wing veins by 3.0 mL disposable syringe and were stored either in vacutainer containing ethylene di-amine tetra acetic acid or in Flinders Technology Associates (FTA) classic card. The vacutainers and classic cards were then labeled with the name of chicken variety, age and sex of the birds and date of collection. The genomic DNA from whole blood samples and FTA card was extracted using both DNA isolation kit (Jena bioscience, Germany and GeNet Bio, Daejeon, Korea) and phenol-chloroform method.

### Polymerase chain reaction amplification and genotyping

#### Selection of primers and polymerase chain reaction amplification

Sixteen (16) highly polymorphic MS markers were selected from the list of markers previously reported in Cho et al [15], which were selected from the ark database (http://www.thearkdb.org/using-arkdb/). Forward primers were labelled and modified with four types of fluorescence dye. The detailed information for primers used in this study is mentioned in Cho et al [15]. Multiplex polymerase chain reaction (PCR) for total of 161 DNA samples was performed in a 20 μL reaction volume. The PCR master mixture was consisting of 2.0 μL of 25 ng/μL gDNA, 10 μL of HS Prime multiplex PCR buffer (GeNet Bio, Korea) and 0.5 μL of 5 pmol forward and reverse primer and appropriate volume of triple distilled water to adjust the total volume. PCR was performed in an initial denaturation at 94°C for 10 min followed by 35 cycles of denaturation at 94°C for 30 s, annealing temperature at 60°C for 30 s, extension temperature at 72°C for 30 s and final extension at 72°C for 20 min using the C1000 Thermal Cycler (Bio-Rad, Irvine, CA, USA). PCR amplifications products were confirmed by 2.0% agarose gel electrophoresis.

#### Microsatellite genotyping

Fragment analysis was performed by capillary electrophoresis array using the Genetic analyser 3,130 xl (Applied Biosystems, Foster City, CA, USA). The genotyping reaction was performed in a 10 μL total volume including 1.0 μL of diluted PCR product, 10 μL of Hi-Di formamide (Applied Biosystems, USA) and 0.1 μL of the GeneScan-500LIZ size standard (Applied Biosystems, USA). The genotyping results were obtained using GeneMapper software ver. 3.7 (Applied Biosystems, USA).

### Data analysis

At first raw data were checked using MS tool kit (in excel) for detection of any genotyping error and null alleles. The evidence for significant deviation from population mutation-drift equilibrium (bottleneck) at 16 neutral MS markers in all populations was checked after Bonferroni correction, with 2,000 replicates under the two phase mutation model with 95% step-wise mutations (Ps = 0.95) using BOTTLENECK program [16]. Measures of genetic diversity such as total number of alleles, allele frequencies, mean number of alleles (MNA), polymorphism information content (PIC), observed and expected heterozygosity (*Ho*, *He*) and fixation index (F) were computed using GenAlEx ver.6.5 [17]. Nei genetic distances were computed using the aforesaid software. Allele richness (AR) was calculated using FSTAT 2.9.3 [18]. Pairwise genetic differentiation (Wright’s F statistics) and analysis of molecular variance (AMOVA) analysis were performed using Arlequin ver. 3.5 [19]. Neighbor-Joining phylogenetic tree was constructed based on Nei’s standard genetic distance model with 1,000 bootstraps using the software POPTREE2 [20]. Genetic structure of the studied chicken populations was inferred by model-based clustering using STRUCTURE ver. 2.3.4 [21] with a burn period of 20,000 generations and Markov Chain Monte Carlo simulations of 100,000 iterations. The optimal K values were determined having the lowest cross-validation (CV) errors (ΔK = 3.74). Discriminant analysis of principal component (DAPC) analysis in R, aims to provide an efficient description of genetic clusters using a few synthetic variables. These are constructed as linear combinations of the original variables (alleles) which have the largest between-group variance and the smallest within-group variance [22].

## RESULTS AND DISCUSSION

### Distribution and morphological features of the experimental birds

Table 1 represents the morphological characteristics and distribution of the investigated chicken samples of Bangladesh. ND and NN are scattered throughout the country except in the hilly areas. Besides, HI chickens are predominantly available in Chittagong Hill tracts. The AS is mostly concentrated in Sarail Upazila of Brahmanbaria district as well as in the peri-urban and urban areas of Dhaka, Chittagong, and Sylhet division. All birds possess single comb type with few exceptions in AS chicken which have a strawberry comb. All the chicken varieties have whitish and/or yellowish shank color except red jungle fowl that possesses slate/blackish shank color. Bhuiyan et al [4], Faruque et al [23] and Sarker et al [24] previously reported the morphological features and geographic distribution of Bangladeshi indigenous chickens that support the present findings.

### Genetic diversity measures within and among the populations

In total, 171 alleles were detected from 16 polymorphic markers (Table 2). The 16 MS markers had an average of 10.7 alleles where the mean *Ho*, *He*, and polymorphism information content (PIC) were 0.669, 0.71 and 0.749, respectively. The ADL0259 marker had the highest number of alleles (16) while ADL0268 possessed the lowest number of alleles (6). The PIC values of locus were ranged between 0.598 (ROSS013) and 0.843 (MCW104) among the studied loci (Table 2). No evidence was observed for possible bottleneck effect in any of the populations using 16 loci. At marker level diversity, the present results are in agreement with the findings of Seo et al [25] who reported that the *Ho*, *He*, and PIC ranged from 0.709 to 0.882, 0.466 to 0.852, and 0.648 to 0.865, respectively in Korean native chicken lines. On the other hand, considering five chicken populations of Bangladesh, the overall MNA was 7.06±0.45 (Table 3). The lowest MNA was found in JF (5.81±0.46) whereas the highest number of alleles was found in ND (7.13±0.52). The highest Shanon’s information index, an estimator of diversity index ranged between 1.40±0.08 and 1.54±0.08 in ND and AS, respectively. The average *Ho* was 0.67±0.01 among the investigated populations. However, the highest *Ho* was found in NN (0.71±0.03) and the lowest heterozygosity (0.64±0.03) possessed by ND and HI chicken populations. On the other hand, the average Nei’s unbiased *He* was 0.70±0.01 for all loci and varied between 0.68±0.03 and 0.70±0.03 in ND and NN chicken populations, respectively (Table 3). The overall fixation index (*F*) was found 0.04±0.02 for all loci with a range between −0.02 ±0.02 (NN) and 0.08±0.03 (JF).The findings of the current study on MNA, *Ho*, and *He* are in concordance with Chinese, Ethiopian and Tanzanian chicken populations reported in several literatures[13,26–28]. However, the MNA observed in the present study were lower than those reported values of Pirany et al [9] and Dorji et al [7]. They found the average MNA were 10.33±4.33 and 14.17±0.93, respectively, in Indian and Bhutanese chicken populations. By contrast, Van Marle-Köster and Nel [11] reported relatively lower MNA than the current findings that ranged from 2.3 to 4.3 in five African chicken lines.

Comparing to the present results on *Ho* and *He*, Rudresh et al [29] reported higher values (0.69 to 0.86) in two chicken populations of Karnataka, India while lower estimates (0.44 to 0.58) were found in Thai and Bhutanese native chickens [7]. It is notable to mention that direct comparison of data from different studies is probably difficult due to the different genetic backgrounds of the chicken populations studied and the different MS markers used [26]. The differences in MNA, *Ho*, and *He* between previous and present findings may be attributed with sample size, population structure, number of markers used, population specific alleles and/or allele scoring bias (null allele or allele drop out) and sampling strategy. The higher MNA, and mean allele richness values estimated based on the minimum sample size 17 and weighted over 16 loci indicated relatively higher genetic diversity in all populations and therefore, Bangladeshi chicken populations are more diverse. Markers with PIC values >0.5 and *He* values >0.6 provided high PIC for genetic diversity measures and were most reliable for population discrimination [25,30]. The present study confirmed that all markers had higher PIC and *He* values than the truncated level, except for ADL0304. Taken together, the observed high heterozygosity value, PIC, Shanon’s Information Index (Tables 2, 3) indicated that the selected 16 MS markers were reliable and informative for estimating the genetic diversity in indigenous chicken population of Bangladesh.

According to Weir and Cockerham [31] estimation, the overall within population inbreeding (F_IS_) was found to be 0.046 considering all 16 loci and varied between −0.07 (LEI 0268 locus) and 0.14 (ADL0259 locus) (Table 3). The overall heterozygote deficiency or total inbreeding (F_IT_) and mean genetic distance (F_ST_) were 0.16 and 0.12, respectively, over the 16 loci. Seo et al [25] discriminated five Korean native chicken lines using 15 highly polymorphic MS markers where the estimated mean F_IS_, F_IT_, and F_ST_ values were 0.0093, 0.137, and 0.129, respectively and is comparable to the present study. Furthermore, Chen et al [27] reported the mean F_IS_ and F_IT_ values to be 0.002 and 0.18, respectively, in fifteen Chinese Indigenous chicken breeds and are comparable to the present study. The F_IS_ represents a degree of non-random mating (deviation from Hardy-Weinberg equilibrium) where a positive value for F_IS_ means selective breeding population. Only five (MCW0123, LEI0094, ADL0293, ADL0268, ROS0083) out of 16 markers showed a negative number. This result indicated the absence of random mating in the investigated chicken populations, which was supported by Faruque et al [8] who reported that three indigenous chicken varieties (ND, NN, and HI) were under selective breeding for a decade at the institutional flocks. Consequently, the rate of inbreeding has been increased over the generations. In addition, the higher F_IT_ value of the present study was due to excessive homozygosity existing in six MS markers (MCW 0029, MCW0264, ROS0013, ADL0304, LEI0074, and LEI 0141).

### Genetic distances and clustering of the indigenous chicken varieties

#### Genetic distances

**Figure 1:**
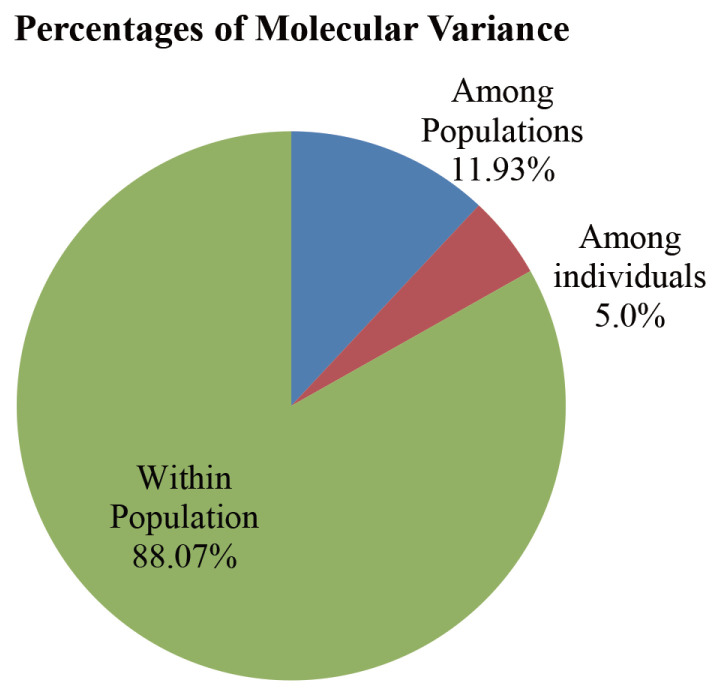
Analysis of molecular variance calculated based on the allelic distance matrix of F_ST_ statistics among five chicken populations of Bangladesh. F-statistics were significant at p<0.05.

The populations pairwise distances (F_ST_) among the studied populations were found significant (p< 0.01) and the values ranged between 0.084 and 0.154 (Table 4). The highest pairwise genetic distance (0.154) was found between ND and AS while the lowest distance was observed between JF and AS (0.084). Nei’s unbiased genetic distance results were also in agreement with pairwise genetic distances showing highest distance (0.578) between ND and AS, whereas the lowest genetic distance (0.285) was between AS and JF. The AMOVA results showed the genetic variation among the 5 populations was 11.93% while the remaining 88.07% of the total variation was accounted for within population diversity with an overall F_ST_ value of 0.12 (p<0.001) (Figure 1). Several previous studies reported higher F_ST_ values (0.15 to 0.26) in Indian [14], Chinese [27] and African indigenous chicken populations [10] and depicted that comparatively lower genetic distances existed among the Bangladeshi chicken varieties. On the contrary, Halima et al [26] reported the genetic distances in seven North-west Ethiopian native chicken populations varied from 0.073 to 0.13. Seo et al [25] found pairwise genetic distance among five Korean native chicken lines to be 0.083 to 0.171. The above two findings are similar to the present study. In addition, the current results are supported by the study of Yamamoto et al [32] who reported the lower genetic differentiation among the different Bangladeshi chicken populations. Bhuiyan et al [5] found low F_ST_ value (0.1084) for Bangladeshi chicken varieties using mtDNA D-loop sequence analysis and strongly justified the results of this study as evidenced by population pairwise F_ST_ and AMOVA analysis. Altogether, the low genetic differentiation among the Bangladeshi chicken populations might be due to recent gene flow among themselves as well as common ancestor for constructing the populations.

#### Phylogenetic analysis

**Figure 2:**
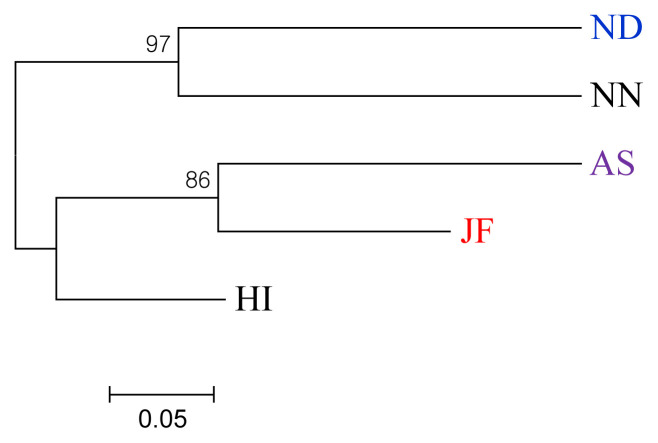
Neighbor-Joining phylogenetic tree based on standard genetic distance (Nei, 1972) depicting relationships among five indigenous chicken populations of Bangladesh. ND, non-descript Deshi; NN, naked neck; HI, hilly; JF, red jungle fowl; AS, Aseel.

The Nei’s unbiased genetic distance matrices and dendrogram have shown that NN and ND were grouped into the same branch (Table 4, Figure 2). The three other chicken varieties AS, HI, and JF made a separate cluster which revealed the close relatedness among themselves. Notably, HI was separated from AS and JF with a node that signifies HI as a distinct chicken variety. Bhuiyan et al [4] and Faruque et al [8] reported ND, NN, and HI were distinct indigenous chicken varieties of Bangladesh where HI are geographically isolated from others and supports the present findings. This phenomenon could also be explained from the geographic history, as both JF and HI are the inhabitants of Chittagong Hill Tract regions and might be separated from the common origin in the recent past. AS is an established fighting breed but has a close relationship with JF. This might be due to the fact of *inter se* mating between AS and JF, a long tradition to the farmers, for increasing vigor and aggressiveness in AS birds (personal communication). The phylogenetic tree also suggested the possible introgression of JF to AS breed and compatible with the previous mitochondrial DNA study [5]. Besides, ND and NN are evenly distributed throughout the country. Gene flow between these two populations is not unlikely and therefore, they belong to the same cluster due to their close genetic relationships.

**Figure 3:**
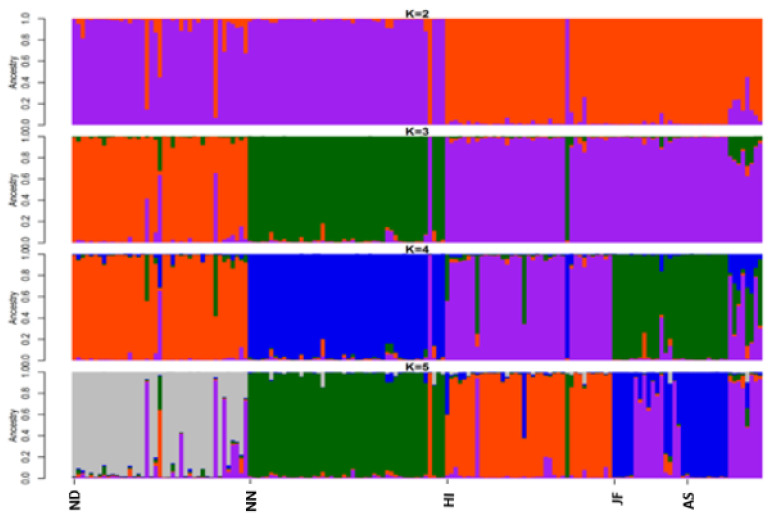
Structure clustering of indigenous chicken varieties among 5 different chicken populations of Bangladesh. The proportions of ancestral populations for each individual varying from K = 2 to 5 for population structure construction. ND, non-descript Deshi; NN, naked neck; HI, hilly; JF, red jungle fowl and AS, Aseel.

**Figure 4:**
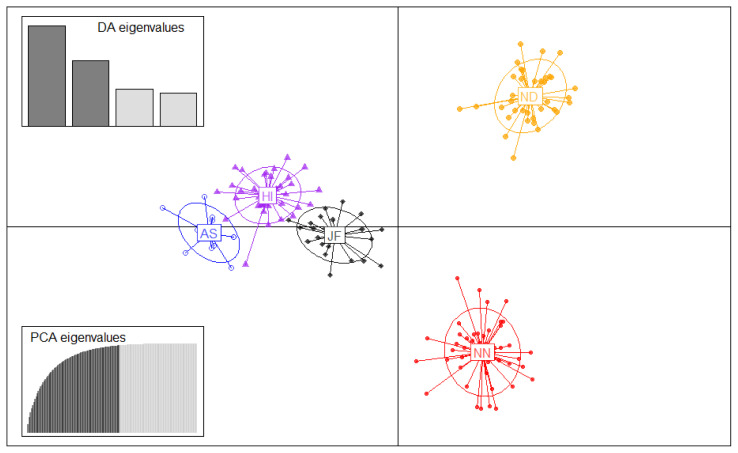
Discriminate analysis of principal component (DAPC) results using five different indigenous chicken population of Bangladesh. DAPC aims to provide an efficient description of genetic clusters using a few synthetic variables constructed as linear combinations of the original variables (alleles).

Structure program was employed to investigate the genetic structure of the 5 chicken populations of Bangladesh (Figure 3). The results showed better agreement in structure output at K values between 4 and 5. The output at K = 5 seems plausible; it clearly distinguished each chicken population. This result is supported by the findings of Cho et al [15] and Seo et al [25] where they reported Korean native chicken breeds (five or six) had an equal number of underlying genetic clusters (K = 5 or 6). The ΔK statistic was 3.74 at a maximum K value (K = 5), which suggest the most probable number of inferred clusters. Structure data also indicated a certain portion of genetic admixture in each population from other varieties. Moreover, structure analysis also depicted remarkable introgression of JF into AS, which was supported by the findings of Bhuiyan et al [5] and Sarker et al [24]. K-means based genetic clustering in DAPC analysis was given at K-mean = five (lowest BIC value) and group membership values (data not shown) were consistent with structure results. Efficient description of genetic clusters among Bangladesh chicken illustrated in DAPC plot (Figure 4) also provides membership probabilities of individuals for the different groups based on the retained discriminant functions. DAPC result was similar to structure results indicating that all groups were discriminated, however, a close proximity between AS, JF, and HI was observed. It is noteworthy to mention that the genetic basis of the founder population of these five chicken varieties was not well defined as well as a bit complicated. Eggs were screened from the farmers merely based on phenotypic information from the random mated population. They were being reared in scavenging condition and therefore, some gene flow in neighbouring regions or populations possibly exists. Taken together, this study provides important information on genetic background of indigenous chicken genetic resources of Bangladesh that could be utilized for conservation and subsequent improvement.

## IMPLICATIONS

The molecular study showed that indigenous chicken of Bangladesh still possesses rich genetic diversity. Analysis of molecular variance analysis revealed only 11.93% of the total genetic diversity accounted for between population differentiations (F_ST_) that indicates poor genetic variability among the investigated populations. The phylogenetic tree and discriminant analysis of principal component analysis showed hilly chicken appeared to be genetically closer to Aseel and red jungle fowl. Structure analysis depicted a certain amount genetic admixture among the five studied populations where massive introgression was found from red jungle fowl to Aseel. Altogether, this study provides some important insight information for the first time on genetic diversity measures and population structure inferences of Bangladeshi chicken populations using microsatellite markers.

## Figures and Tables

**Table 1:** Morphological features of Bangladeshi chicken populations and JF used in this study

Type/variety 1)	No. of sample	Distribution	Morphological characteristics		

			Comb type	Plumage	Shank
ND	41	Throughout the country	Single	Reddish brown or reddish black	White, yellow, black
HI	39	Chittagong hill tract region	Single	Black dotted on white and Grey or reddish	Whitish, yellow, black
NN	46	Throughout the country	Single	Black and/or reddish black	White, yellow, black
AS	18	SarailUpazila of Brahmanbaria district and peri-urban and urban areas of Dhaka, Chittagong and Sylhet division	76%-pea comb; 24% strawberry comb	Deep purple	Yellow
JF	17	Hilly areas of the Chittagong region and in Sundarban (the largest mangrove forest)	Single	Mixed feather colors with orange, brown, red, gray, white, and even metallic green plumage	Blackish/slate color

Source: Bhuiyan et al [4]; Faruque et al [23]; Sarker et al [24].

1) ND, non-descript Deshi; HI, hilly; NN, naked neck; AS, Aseel; JF, jungle fowl.

**Table 2:** Polymorphism information and F- statistics of microsatellite markers used in this study

Locus	K	*Ho*	*He*	PIC	F_IS_(f)	F_IT_(F)	F_ST_(θ)	HW
MCW0123	8	0.659	0.639	0.671	−0.018	0.108	0.123	NS
ADL0317	10	0.725	0.758	0.784	0.017	0.110	0.095	NS
MCW0087	10	0.680	0.749	0.791	0.063	0.169	0.113	*
ADL0259	16	0.699	0.757	0.783	0.135	0.213	0.090	***
LEI0094	15	0.717	0.724	0.829	0.008	0.210	0.204	**
ADL0293	9	0.722	0.711	0.704	−0.005	0.062	0.067	NS
MCW104	13	0.728	0.801	0.843	0.052	0.128	0.080	***
MCW0330	7	0.688	0.703	0.741	0.001	0.125	0.125	NS
ADL0268	6	0.689	0.647	0.697	−0.065	0.145	0.197	NS
LEI0141	9	0.613	0.700	0.729	0.123	0.205	0.093	*
ROS0083	10	0.623	0.609	0.613	−0.029	0.047	0.074	NS
LEI0074	8	0.648	0.744	0.782	0.088	0.192	0.113	***
ADL0304	10	0.644	0.722	0.747	0.089	0.194	0.115	NS
ROS0013	10	0.492	0.563	0.598	0.106	0.233	0.142	**
MCW0029	15	0.701	0.749	0.835	0.08	0.226	0.159	***
MCW0264	15	0.669	0.767	0.836	0.092	0.208	0.128	NS
Mean	10.7	0.669	0.71	0.7489	0.046	0.161	0.120	-

K, number of alleles in all population; *Ho*, observed heterozygosity per locus; *He*, expected heterozygosity; PIC, polymorphic information content; Weir and Cockerham [31] estimation of F_IT_ (F), F_IS_ (f) and F_ST_ (θ); HW, Hardy-Weinberg equilibrium test (with Bonferroni correction).

**Table 3:** Genetic diversity measures of 5 chicken populations using 16 polymorphic markers

Population1)	N	Na	Range	I	AR	*Ho*	*He*	F
ND	41	7.13±0.52	5–11	1.40±0.08	5.76	0.64±0.03	0.67±0.03	0.04±0.03
NN	46	6.81±0.51	4–11	1.43±0.06	5.58	0.71±0.03	0.70±0.02	−0.02±0.02
HI	39	6.56±0.47	3–10	1.44±0.08	5.78	0.64±0.03	0.69±0.02	0.07±0.04
JF	17	5.81±0.46	3–10	1.41±0.06	5.73	0.65±0.03	0.70±0.02	0.08±0.03
AS	18	7.06±0.45	4–11	1.54±0.08	6.82	0.70±0.04	0.72±0.03	0.02±0.05
Overall mean	-	7.06±0.45	-	1.44±0.03	5.93	0.67±0.01	0.70±0.01	0.04±0.02

N, No. of observation; Na, number of alleles; I, Shanon’s information index; AR, allele richness; *Ho*, observed heterozygosity; He, expected heterozygosity; F, fixation index across loci for each population.

1) ND, non-descript Deshi; HI, hilly; NN, naked neck; JF, jungle fowl; AS, Aseel.

**Table 4:** Population pairwise F_ST_ (above diagonal) and Nei’s unbiased genetic distance (below diagonal) for five chicken populations of Bangladesh

Items	ND	NN	HI	JF	AS
ND	-	0.125**	0.112**	0.139**	0.154**
NN	0.391	-	0.118**	0.123**	0.138**
HI	0.337	0.390	-	0.091**	0.092**
JF	0.475	0.429	0.292	-	0.082**
AS	0.578	0.529	0.305	0.285	-

ND, non-descript Deshi; NN, naked neck; HI, hilly chicken; JF, jungle fowl; AS, Aseel.

** p<0.05.
